# New Medical Journal

**Published:** 1853-02

**Authors:** 


*New Medical Journal.*—A new Medical Journal has been commenced at Nashville, Tenn. It is entitled the *Southern Journal of the Medical and Physical Sciences*, and is edited by Drs. Jno. W. Sting, Wm. P. Jones, R. O. Cuney, and B. Wood. It is to embrace subjects pertaining to dentistry. It is a bi-monthly of seventy-two pages. The typographical appearance is good, and as it has four editors, it is to be presumed there will be no lack of original matter. We wish the enterprize success.

